# Coenzyme Q10 Regulation of Apoptosis and Oxidative Stress in H_2_O_2_ Induced BMSC Death by Modulating the Nrf-2/NQO-1 Signaling Pathway and Its Application in a Model of Spinal Cord Injury

**DOI:** 10.1155/2019/6493081

**Published:** 2019-12-12

**Authors:** Xing Li, Jiheng Zhan, Yu Hou, Yonghui Hou, Shudong Chen, Dan Luo, Jiyao Luan, Le Wang, Dingkun Lin

**Affiliations:** ^1^Department of Orthopedic Surgery, The Second Affiliated Hospital of Guangzhou University of Chinese Medicine, No. 111 Dade Road, Guangzhou, Guangdong 510120, China; ^2^Guangzhou University of Chinese Medicine, No. 12, Jichang Road, Baiyun District, Guangzhou 510405, China; ^3^Lingnan Medical Research Center of Guangzhou University of Chinese Medicine, Guangzhou 510405, China; ^4^Department of Spine Surgery, The First Affiliated Hospital of Sun Yat-sen University, Guangzhou, China

## Abstract

Spinal cord injury (SCI) has always been considered to be a devastating problem that results in catastrophic dysfunction, high disability rate, low mortality rate, and huge cost for the patient. Stem cell-based therapy, especially using bone marrow mesenchymal stem cells (BMSCs), is a promising strategy for the treatment of SCI. However, SCI results in low rates of cell survival and a poor microenvironment, which limits the therapeutic efficiency of BMSC transplantation. Coenzyme Q10 (CoQ10) is known as a powerful antioxidant, which inhibits lipid peroxidation and scavenges free radicals, and its combined effect with BMSC transplantation has been shown to have a powerful impact on protecting the vitality of cells, as well as antioxidant and antiapoptotic compounds in SCI. Therefore, we aimed to evaluate whether CoQ10 could decrease oxidative stress against the apoptosis of BMSCs *in vitro* and explored its molecular mechanisms. Furthermore, we investigated the protective effect of CoQ10 combined with BMSCs transplanted into a SCI model to verify its ability. Our results demonstrate that CoQ10 treatment significantly decreases the expression of the proapoptotic proteins Bax and Caspase-3, as shown through TUNEL-positive staining and the products of oxidative stress (ROS), while increasing the expression of the antiapoptotic protein Bcl-2 and the products of antioxidation, such as *glutathione* (GSH), against apoptosis and oxidative stress, in a H_2_O_2_-induced model. We also identified consistent results from the CoQ10 treatment of BMSCs transplanted into SCI rats *in vivo*. Moreover, the Nrf-2 signaling pathway was also investigated in order to detail its molecular mechanism, and the results show that it plays an important role, both *in vitro* and *in vivo*. Thus, CoQ10 exerts an antiapoptotic and antioxidant effect, as well as improves the microenvironment *in vitro* and *in vivo*. It may also protect BMSCs from oxidative stress and enhance their therapeutic efficiency when transplanted for SCI treatment.

## 1. Introduction

Spinal cord injury (SCI) has always been considered as a devastating problem that results in catastrophic dysfunction, high disability rate, low mortality rate, and huge cost for the patients. It not only affects the physical and mental health of patients but is also an economic burden on the family [1–3]. At present, although various pharmacological therapies are used for SCI, functional recovery is still uncertain [4–6].

Recently, stem cell-based therapy, especially with the use of bone marrow mesenchymal stem cells (BMSCs), has been shown to be a promising SCI therapy strategy [7–9]. Numerous previous studies have illustrated the manner by which BMSC therapy in a dose-dependent manner improves the functionality in rat, rabbit, monkey, and even human models of SCI [8, 10–12]. However, low cell survival rates in a poor microenvironment have been observed after SCI treatment using BMSCs, which limit its therapeutic efficiency [13]. Oxidative stress is a critical factor that contributes to the development of a poor microenvironment during the progression of SCI [14, 15]. Therefore, methods that can be used to decrease oxidative stress, reduce apoptosis, and increase the vitality of the transplanted BMSCs are important factors that need to be researched in order to develop efficient treatment strategies for SCI [16].

Coenzyme Q10 (CoQ10) is known to be a powerful antioxidant, which inhibits lipid peroxidation and scavenges free radicals [17, 18]. Under treatment of SCI, some studies have shown that CoQ10 reduces the level of oxidative injury and antiapoptosis [19–22]. Medication combined with BMSC transplantation has been shown to have a powerful impact on protecting the vitality of cells and antioxidant and antiapoptotic compounds in SCI [23, 24]. However, the method by which CoQ10 treatment can be combined with BMSC transplantation in SCI is still unclear. Therefore, in this study, we aimed to evaluate whether CoQ10 could decrease oxidative stress and prevent the apoptosis of BMSCs *in vitro*, as well as explore its molecular mechanisms. Furthermore, we investigated the protective effect of CoQ10 combined with BMSC transplantation in a SCI model to verify its viability.

## 2. Materials and Methods

### 2.1. Materials

Male Sprague Dawley (SD) rats were supplied by the Guangzhou University of Chinese Medicine (Guangzhou, China). All procedures were performed in accordance with the animal guidelines of the Guangzhou University of Chinese Medicine. The study was approved by the ethics committee of Guangzhou University of Chinese Medicine. *α*-Modified Eagle's medium (*α*-MEM), fetal bovine serum (FBS), and trypsin were purchased from Gibco-BRL (NY, USA). A WST-1 cell proliferation and cell viability detection kit was obtained from Nanjing KeyGen Biotech Co. (Nanjing, China). Malondialdehyde (MDA), superoxide dismutase (SOD), and glutathione (GSH) assays were purchased from Nanjing Jiancheng Bioengineering Institute (Nanjing, China). CoQ10 (purity > 98% using HPLC) was purchased from Sigma-Aldrich (St. Louis, MO, USA), dissolved in DMSO (St. Louis, MO, USA), and diluted in a culture medium. The final concentration of the DMSO was 0.1%.

### 2.2. Cell Isolation and Treatment

Rat BMSC isolation was performed by collecting the cells from the bone marrow of the femurs and tibiae of 4-week-old rats, as previously described [25]. The BMSCs were plated into culture dishes containing *α*-MEM supplemented with 10% FBS, at 37°C in a humidified 5% CO_2_ environment. The culture medium was changed every other day. BMSCs from passages 3 to 5 were used for all experiments. The cells were pretreated with CoQ10 for 2 h and then cotreated with H_2_O_2_ (300 *μ*M) for 24 h.

### 2.3. Immunophenotypical Analysis of BMSCs

BMSCs at a density of 3 × 10^5^ cells were used for the phenotypic analysis using flow cytometry [26]. In brief, the cells were stained with phycoerythrin- (PE-) labeled primary antibodies against CD11, CD73, and the corresponding isotype control antibody of rat IgG1, in the dark at room temperature, and then fixed in 1% paraformaldehyde (PFA), after being washed twice with ice-cold PBS. Surface maker expression and the purity of the cells were detected using flow cytometry.

### 2.4. Differentiation of the BMSCs

In order to analyze whether the culture of BMSCs possesses multipotent differentiation ability *in vitro*, passage 3 BMSCs were cultured in a chondrogenic induction medium containing 0.3% ascorbic acid, 0.01% dexamethasone, 0.1% sodium pyruvate, 1% insulin-transferrin-selenium+premix, 0.1% proline, and 1% TGF-*β*3 or an osteogenic induction medium containing 1% *β*-glycerophosphate, 0.01% dexamethasone, and 0.2% ascorbic acid for 2 weeks. The induction medium was changed every 3 days. After differentiation, the cells were fixed with 4% PFA for 20 min for alkaline phosphatase staining, alizarin red staining, and alcian blue staining. After being washed three times in PBS, representative photographs were captured under a microscope.

### 2.5. Cell Viability Assay

Cell viability was evaluated using a WST-1 assay [27]. In brief, BMSCs were seeded into a 96-well plate at a density of 1 × 10^4^/well. The cells were then pretreated with various concentrations of CoQ10 for 2 h, followed by coincubation with H_2_O_2_ for 24 h. After incubation, 10 *μ*l of WST-1 solution was added into each well of the plate and left to incubate for another 2 h at 37°C. Absorbance was then measured at a wavelength of 450 nm, using a microplate reader (Bio-Rad, USA). All experiments were performed in triplicate.

### 2.6. LDH Assay

Cell cytotoxicity was determined using a LDH assay kit (Nanjing Jiancheng Bioengineering Institute, Nanjing, China). LDH was released into the culture medium as a result of cell membrane intact damage caused by H_2_O_2_. After treatment, the cell culture medium was analyzed as instructed by the manufacturer. Absorbance was measured at 450 nm, using a microplate reader (Bio-Rad, USA). All experiments were performed in triplicate.

### 2.7. Flow Cytometric Evaluation of Apoptosis

After treatment, the apoptotic cells were fluorescently labeled using an Annexin V-FITC apoptosis detection kit (Roche Applied Science, Basel, Switzerland), by following the manufacturer's instructions. In brief, BMSC pellets were collected and washed twice with cold PBS and gently resuspended in 500 *μ*l of a binding buffer containing 5 *μ*l of Annexin V-FITC and 5 *μ*l of PI for 15 min at room temperature. Then, the samples were analyzed using flow cytometry (BD Biosciences).

### 2.8. TUNEL Assay

The terminal deoxynucleotidyl transferase dUTP nick end labeling (TUNEL) assay was performed using a TUNEL cell apoptosis detection kit (Roche Applied Science, USA), by following the manufacturer's instructions. DAPI was used to stain the nuclei. The results were captured using fluorescence microscopy (Olympus, Japan).

### 2.9. RT-qPCR Analysis

After treatment, total RNA was extracted using a TRIzol reagent (Invitrogen, USA), by following the manufacturer's instructions. The First-Strand cDNA Synthesis Kit along with a PrimeScript RT reagent kit (TaKaRa Biotechnology Co. Ltd., Japan) was used to conduct RT-PCR analysis on a SYBR green system (Toyobo, Japan Ltd., Japan). The primer sequences used are given in Table 1.

### 2.10. Measurement of Oxidative Stress

Intracellular ROS generation was measured using H_2_DCF-DA (Sigma Aldrich), as reported [28], while the level of GSH was determined using a GSH assay kit, by following the manufacturer's instructions.

### 2.11. Western Blotting Analysis

Western blotting experiments were performed following standard methods [27]. In brief, the proteins were loaded onto 10% SDS-PAGE and electrotransferred onto polyvinylidene difluoride membranes (Millipore, USA). The membranes were incubated with primary antibodies against Bax and Caspase-3 (1 : 1,000, Cell Signaling Technology), as well as Bcl-2, NQO-1 (1 : 1000 dilution, Abcam, UK), and Nrf-2 (1 : 1,000, R&D), overnight at 4°C, followed by sequential incubation with the secondary antibodies conjugated with horseradish peroxidase (HRP) (1 : 1000, Cell Signaling Technology), at room temperature for 2 h. GAPDH (1 : 1000 dilution, Abcam, UK) was used as the internal reference. Quantified densitometric analysis was conducted using an ImageQuant LAS 4000 mini detection system (GE Healthcare, Buckinghamshire, UK), and the results were analyzed using ImageJ software (National Institutes of Health, Bethesda, MD).

### 2.12. Experimental SCI Model and BMSC Transplantation

Fifty adult male SD rats weighing 200-250 g were randomly divided into 5 groups (*n* = 10 per group): (1) the sham group, (2) the SCI model group, (3) the BMSC group, (4) the CoQ10 group, and (5) the CoQ10+BMSC group. A SD rat SCI model was induced with a moderate contusion based on Allen's method, as previously described [29]. In brief, SD rats were anesthetized with sodium pentobarbital (40 mg/kg, i.p.). Then, the incision area was shaved, and a laminectomy was performed at T9-T10 levels, under sterile conditions. After exposing the spinal cord surface with an intact dura, a 10 g weight impactor (diameter, 2 mm) was dropped from a height of 50 mm onto the exposed dura at the T10 level. Successful induction of SCI led to spinal cord congestion, tail swing reflexes, swaying of legs, and slow paralysis. Each rat was provided with bladder pressing 3 times daily. 3 days after SCI induction, the rats in the BMSC group and CoQ10+BMSC group were injected with BMSCs through the tail vein. CoQ10 (20 mg/kg) was orally administered for 2 days before surgery and continued until the rats were sacrificed. Similar procedures and treatments were performed on all vehicle groups. All SD rats were sacrificed 1 week postsurgery.

### 2.13. MDA, SOD, and GSH Assays

Following the manufacturer's instructions for the MDA, SOD, and GSH assays, the liquid supernatant of the spinal tissue samples was obtained for measurement. The MDA levels were determined at 532 nm, the SOD activity was measured at 550 nm, and GSH levels were determined at 420 nm, using a microplate reader (Bio-Rad, USA). All experiments were performed in triplicate.

### 2.14. Immunohistochemical Analysis

The rats were anesthetized and then perfused transcardially using ice-cold PBS followed by 4% PFA for 30 min. A 10 mm segment at the center lesion site of the spine was collected for experiments. The samples were cut into serial crosswise sections with a thickness of 4 *μ*m, using a cryostat microtome (Leica RM2016; Leica Instruments, Heidelberg, Germany). The spine tissues were treated with 3% hydrogen peroxide to block endogenous peroxidase and were incubated with 2% normal goat serum [30]. The sections were incubated overnight with Caspase-3 (1 : 50 in PBS, Cell Signaling Technology) at 4°C. The sections were then washed with PBS and incubated with secondary antibodies. Specific labeling was detected using biotin-conjugated goat anti-rabbit IgG and avidin-biotin peroxidase complex (Vector Laboratories, DBA). Images were captured under fluorescence microscopy (Olympus DP80, Japan). Positive staining quantitative analysis was performed using ImageJ software (National Institutes of Health, Bethesda, MD).

### 2.15. Statistical Analysis

Data are expressed as mean ± standard deviation. Statistical analyses were performed using SPSS version 16.0 software (SPSS Inc., Chicago, IL, USA). One-way analysis of variance (ANOVA) or Student's *t*-test was used to identify differences among groups. A *P* value of <0.05 was considered statistically significant.

## 3. Results

### 3.1. Characterization of BMSCs

**Figure 1:**
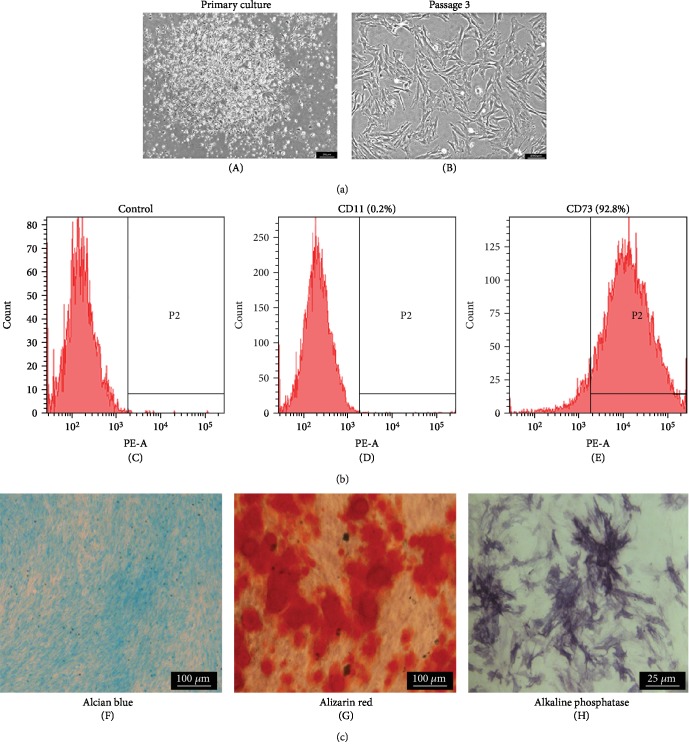
Morphologies, phenotypic characterizations, and differentiation of BMSCs. (a) Representative fields of BMSC morphologies at the primary passage (A) and passage 3 (B). (b) The phenotypic characterizations of BMSCs were identified with the corresponding isotype control (C), CD11 (0.2%) (D), and CD73 (92.8%) (F). (c) The differentiation of BMSC analysis: alcian blue staining (F), alizarin red staining (G), and alkaline phosphatase staining (H).

During the initial phase, BMSCs were found to show a fibroblast-like shape with colonies and floating cells in the culture (Figure 1(a)). The presence of floating cells was completely abolished at passage 3 (Figure 1(b)). The results of the flow cytometry analysis show that 92.8% of cells express CD73 and only 0.2% cells express CD11 (Figures 1(c)–1(e)). The BMSCs were found to have a multipotent ability of differentiating into chondrogenic/osteogenic cells upon induction (Figures 1(f)–1(h)).

### 3.2. Protective Effects of CoQ10 on BMSCs Exposed to H_2_O_2_

**Figure 2:**
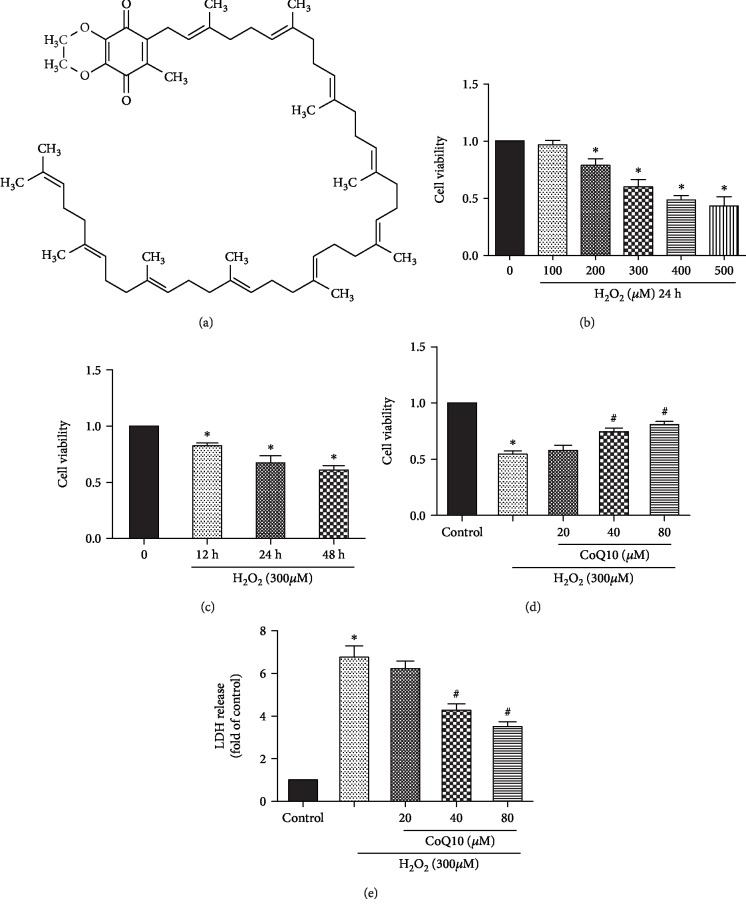
Effects of CoQ10 on the viability of H_2_O_2_-induced BMSCs. Cells were pretreated with different concentrations (20, 40, and 80 *μ*M) of CoQ10 for 2 h followed with H_2_O_2_ (300 *μ*M) for 24 h. (a) Structure of CoQ10. (b) Cells were cultured with different concentrations (100-500 *μ*M) of H_2_O_2_ for 24 h. (c) Cells were cultured with different times (12-24 h) of H_2_O_2_ (300 *μ*M). (d) The cell viability was determined by WST-1 assay. (e) The cell death was determined by LDH assay. Data are presented as mean ± SEM. ^∗^*P* < 0.05 or ^∗∗^*P* < 0.01 compared with the control group. ^#^*P* < 0.05 compared with the H_2_O_2_-induced alone group.

The viability of BMSCs under H_2_O_2_ induction was found to range from 100 to 500 *μ*M within the 24 h period. The chosen concentration of 300 *μ*M H_2_O_2_ was found to result in the death of approximately 50-60% of cells (Figure 2(b)). In order to study the protective effect of CoQ10 on H_2_O_2_-induced BMSC death, WST-1 and LDH assays were performed. The results show that CoQ10 significantly enhances cell viability (Figures 2(c) and 2(d)) and decreases cell death (Figure 2(e)).

### 3.3. CoQ10 Protects BMSCs from Apoptosis Induced by H_2_O_2_

**Figure 3:**
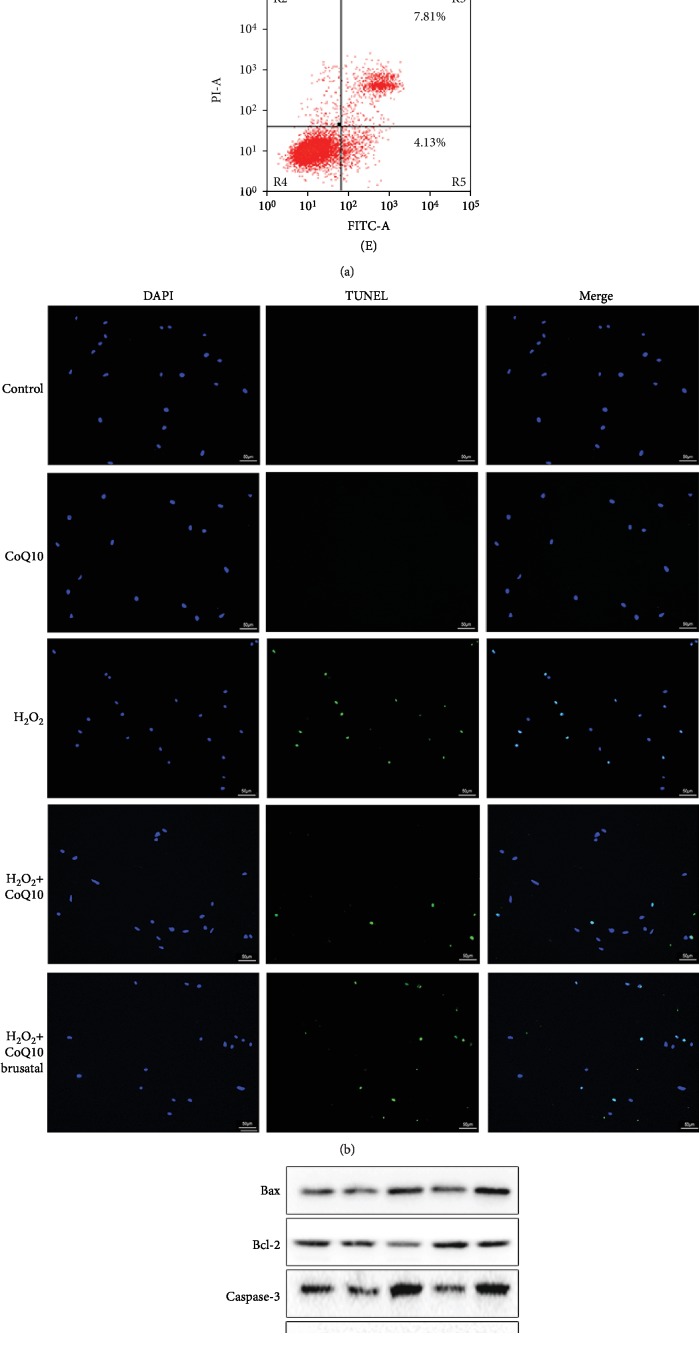
CoQ10 alleviated H_2_O_2_-induced BMSC apoptosis. Cells were pretreated with CoQ10 (40 *μ*M) for 2 h followed with H_2_O_2_ (300 *μ*M) for 24 h. (a) Annexin V-FITC/PI assay was used to evaluate the apoptosis (A) control group, (B) CoQ10 group, (C) H_2_O_2_ group, (D) H_2_O_2_+CoQ10 group, and (E) H_2_O_2_+CoQ10+brusatol group. (b) TUNEL assays (with green fluorescence) measured the cell apoptosis. (c) The protein expression of Bax, Bcl-2, and Caspase-3 was measured by Western blot. (d) The gene expression of Bax, Bcl-2, and Caspase-3 was determined by real-time quantitative PCR. Data are presented as mean ± SEM. ^∗^*P* < 0.05 compared with the control group, ^#^*P* < 0.05 compared with the H_2_O_2_-induced alone group, and ^&^*P* < 0.05 compared with the CoQ10+H_2_O_2_-induced group.

In order to investigate whether CoQ10 decreases H_2_O_2_-induced apoptosis of BMSCs, Annexin V/Propidium Iodide (PI) staining and TUNEL assay were used. The results show that only 4.73% ± 0.36 of Annexin V-FITC and PI-positive stained cells were detected in the control group and 12.78% ± 0.98 of positive cells were detected in the H_2_O_2_ group, while pretreatment with CoQ10 effectively decreased the apoptotic rate to 7.06% ± 0.43 (Figure 3(a)). These results correspond with that of the TUNEL assay (Figure 3(b)). Moreover, upregulation of the proapoptotic proteins Bax and Caspase-3 and downregulation of the antiapoptotic protein Bcl-2 were detected in the BMSCs after H_2_O_2_ induction, both at the protein and mRNA levels (Figures 3(c) and 3(d)), while pretreatment with CoQ10 significantly reversed this expression. Brusatol, a Nfr-2 signal inhibitor, was found to significantly block the protective effect of CoQ10 on H_2_O_2_-induced apoptosis.

### 3.4. CoQ10 Decreases the Antioxidative Effects Exerted on BMSCs

**Figure 4:**
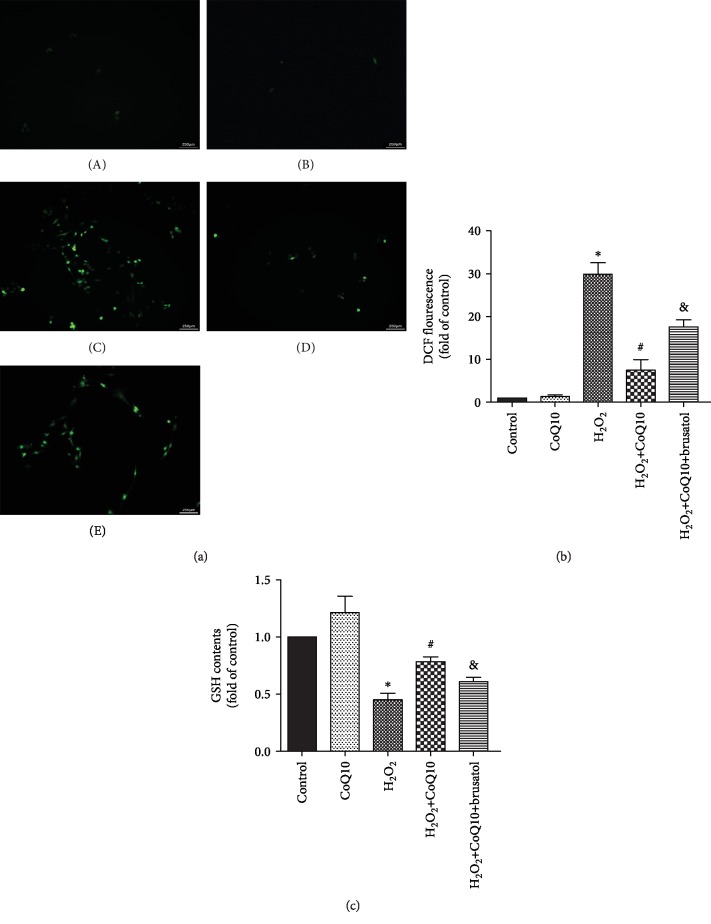
CoQ10 scavenges ROS produced by H_2_O_2_. (a) H2DCF-DA was used to determine the level of ROS induced by H_2_O_2_. (b) Quantitative analysis of DCF fluorescent intensity. (c) GSH assay kit was used to measure the level of GSH. Data are presented as mean ± SEM. ^∗^*P* < 0.05 or ^∗∗^*P* < 0.01 compared with the control group, ^#^*P* < 0.05 compared with the H_2_O_2_-induced alone group, and ^&^*P* < 0.05 compared with the CoQ10+H_2_O_2_-induced group.

In order to confirm the protective effect exerted by CoQ10 on BMSCs as a result of decreased oxidative stress, we evaluated intracellular ROS (Figure 4(a)) and GSH (Figure 4(b)) levels. The results show that the level of ROS significantly increased in the H_2_O_2_-treated group but decreased after CoQ10 treatment. Accordingly, the levels of GSH and brusatol detected confirm the protective effect of CoQ10 on BMSCs against oxidative stress.

### 3.5. CoQ10 Inhibits H_2_O_2_-Induced Apoptosis of BMSCs through the Nrf-2/NQO-1 Signaling Pathway

**Figure 5:**
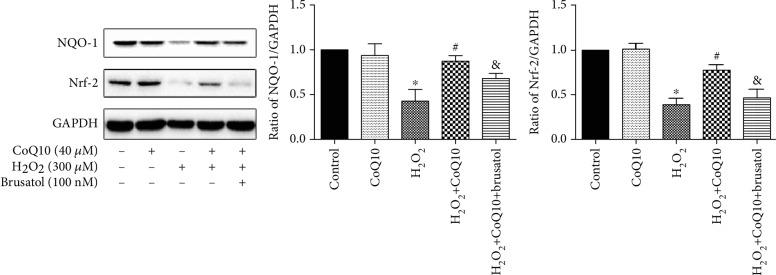
CoQ10 protected BMSCs from H_2_O_2_-induced apoptosis and oxidative stress via the Nrf-2/NQO-1 signaling pathway. Cells were pretreated with CoQ10 (40 *μ*M) for 2 h, followed by coincubation with H_2_O_2_ (300 *μ*M) for 24 h. Data are presented as mean ± SEM. ^∗^*P* < 0.05 compared with the control group, ^#^*P* < 0.05 compared with the H_2_O_2_-induced alone group, and ^&^*P* < 0.05 compared with the CoQ10+H_2_O_2_-induced group.

In order to explore whether the Nrf-2/ARE signaling pathway is involved in the protective effect of CoQ10 against oxidative stress and apoptosis, levels of Nrf-2 and NQO-1 proteins were measured using Western blotting. The results show that protein levels of Nrf-2 and NQO-1 had decreased significantly in the H_2_O_2_-treated group. However, after treatment with CoQ10, this effect was reversed (Figure 5). Furthermore, brusatol was able to partially block the protective effect of CoQ10 during H_2_O_2_-induced cytotoxicity.

### 3.6. Effects of BMSCs, CoQ10, and Their Combined Effect on Oxidative Stress in SCI

**Figure 6:**
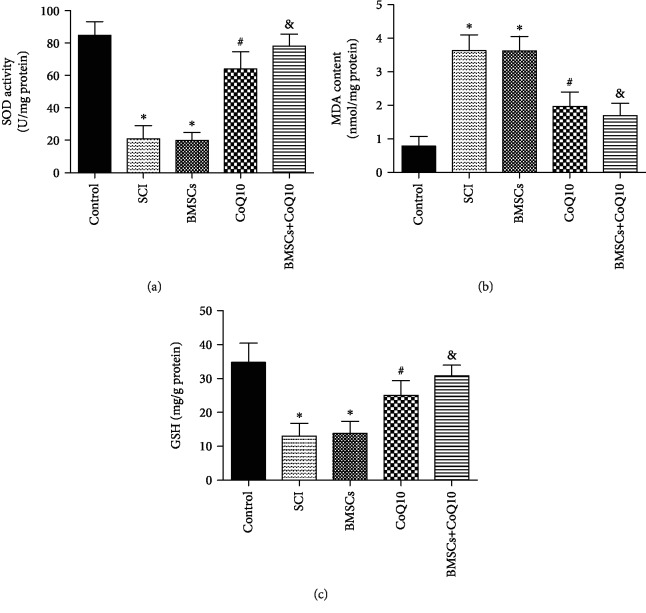
CoQ10 decreased oxidative stress in a rat SCI model. (a) The level of SOD was measured by a SOD assay kit (b) The level of MDA was measured by a MDA assay kit. (c) The level of GSH was measured by a GSH assay kit. Data are presented as mean ± SEM. ^∗^*P* < 0.05 compared with the control group, ^#^*P* < 0.05 compared with the SCI group, and ^&^*P* < 0.05 compared with the BMSC group (*n* = 6).

In order to evaluate the effect of BMSCs, CoQ10, and their combined effect on oxidative stress in SCI, the liquid supernatant of the spinal tissue samples was analyzed using SOD, MDA, and GSH assay kits. The results show that the expression of SOD and GSH had decreased and that the expression of MDA had increased in the SCI group and BMSC group. The administration of CoQ10 and the resulting combination significantly reversed levels of SOD, MDA, and GSH. These results indicate that treatment with CoQ10 and its combination with BMSCs significantly decrease oxidative stress in SCI (Figure 6).

### 3.7. Effects of BMSCs, CoQ10, and Their Combined Effect on Apoptosis in SCI

**Figure 7:**
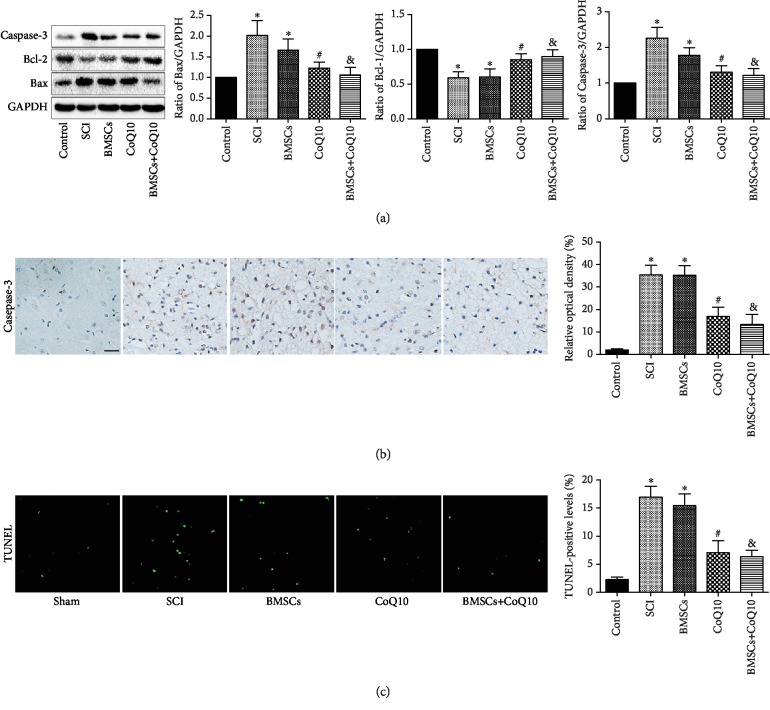
CoQ10 reduced apoptosis in a rat SCI model. (a) The spine tissue sample protein expression of Bax, Bcl-2, and Caspase-3 was measured by Western blot. (b) Immunohistochemical analysis determined the Caspase-3 protein expression. (c) TUNEL staining of the cell apoptosis rate. Scale bar = 50 *μ*m. Data are presented as mean ± SEM. ^∗^*P* < 0.05 compared with the control group, ^#^*P* < 0.05 compared with the SCI group, and ^&^*P* < 0.05 compared with the BMSC group (*n* = 6).

In order to investigate the effects of BMSCs, CoQ10, and their combined effect on apoptosis in SCI, the expression of the proapoptotic proteins Bax and Caspase-3 as well as the expression of the antiapoptotic protein Bcl-2 were detected in the spine tissue samples. The results show that the expression of the Bax and Caspase-3 proteins was upregulated and the expression of the Bcl-2 protein was downregulated in both the SCI group and the BMSC group. The administration of CoQ10 and the resulting combination significantly reversed these results (Figure 7(a)). Moreover, the immunohistochemistry assay also found similar results for the Caspase-3 protein (Figure 7(b)). These findings indicate that treatment with CoQ10 and its combined effect with BMSCs significantly decrease apoptosis in SCI.

### 3.8. Effects of BMSCs, CoQ10, and Their Combined Effect on the Nrf-2/ARE Signaling Pathway in SCI

**Figure 8:**
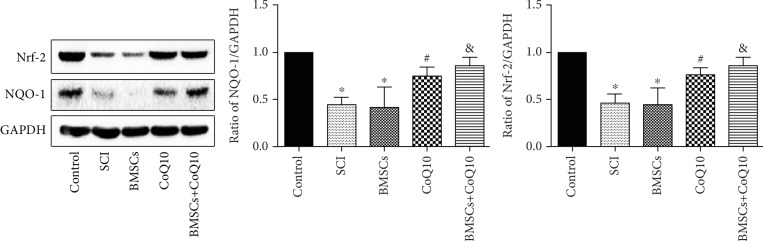
Treatment with CoQ10 combining BMSC transplantation affects the Nrf-2/NQO-1 signaling pathway after SCI. The spine tissue sample protein expression of Nrf-2 and NQO-1 was measured by Western blot. Data are presented as mean ± SEM. ^∗^*P* < 0.05 compared with the control group, ^#^*P* < 0.05 compared with the SCI group, and ^&^*P* < 0.05 compared with the BMSC group (*n* = 6).

In order to examine whether the protective effects of BMSCs, CoQ10, and their combined effect on SCI are activated through the Nrf-2/NQO-1 signaling pathway, the levels of Nrf-2 and NQO-1 proteins in the spine tissue samples were measured. As shown in Figure 8, the protein levels of Nrf-2 and NQO-1 were found to have significantly decreased in both the SCI group and the BMSC group. However, these effects were found to be significantly reversed after the administration of CoQ10 and its combination with BMSCs (Figure 8). These findings indicate that the protective effect of BMSCs, CoQ10, and their combined effect in SCI is activated through the Nrf-2/NQO-1 signaling pathway.

## 4. Discussion

Spinal cord injury (SCI) is one of the most devastating diseases and is still an unresolved event in medicine [2, 31, 32]. A large number of studies have already proven that treatment with BMSCs, which have an ability to differentiate into the neuronal-like cells, is an effective therapy for SCI [23, 33–35]. However, the survival rate of BMSCs after transplantation is low, as a result of the poor microenvironment produced in SCI, which limits its therapeutic efficiency. In the SCI microenvironment, oxidative stress plays a pivotal role and can lead to cellular damage and even cell apoptosis [36, 37]. In our present study, we demonstrate that CoQ10 exerts antioxidative and antiapoptotic effects in H_2_O_2_-induced oxidative injury, which imitates the poor microenvironment of SCI *in vitro*. In the H_2_O_2_-induced model, CoQ10 treatment was found to significantly decrease the expression of the proapoptotic proteins Bax and Caspase-3, as determined through TUNEL-positive staining and the products of oxidative stress (ROS), while increasing the expression of the antiapoptotic protein Bcl-2 and the products of antioxidation (GSH) against apoptosis and oxidative stress. We also found similar effects from CoQ10 treatment of BMSCs transplanted into SCI rats, which also showed decreased levels of oxidative products (MDA), Bax, and Caspase-3 and increased activities of antioxidant enzymes (SOD) and Bcl-2 *in vivo*. Moreover, the Nrf-2 signaling pathway was investigated in detail to reveal its molecular mechanism and the results show that it plays an important role during these processes, both *in vitro* and *in vivo*.

Apoptosis is an important factor of cell inactivation, which is a phenomenon of cell death [38]. Members of the Bcl-2 family of proteins, including Bcl-2 and Bax proteins, play an important role in apoptosis. Bax, the proapoptotic protein, promotes caspase-induced apoptosis by enhancing cytochrome c release [39]. However, Bcl-2, the antiapoptotic protein, inhibits apoptosis by combining with Bax to form a heterodimer that decreases cytochrome c release [28]. Caspase-3, a caspase family member, can cause DNA fragmentation, which ultimately results in cell apoptosis [40, 41]. In our present study, the results show that the upregulation of Bax and Caspase-3 and the downregulation of Bcl-2 are both observed in the H_2_O_2_-induced BMSC model, as well as the rat SCI model. Furthermore, the BMSC transplantation model shows similar results to that of the rat SCI model. These findings are also consistent with that of previous research [24, 25, 42, 43]. However, these results are reversed by CoQ10 treatment, which indicates its antiapoptotic ability. Moreover, terminal deoxynucleotidyl transferase dUTP nick end labeling (TUNEL) staining and immunohistochemistry analysis were also used to further confirm the antiapoptotic effects *in vitro* and *in vivo*, respectively. Together, these results indicate that CoQ10 exerts an antiapoptotic function, both *in vitro* and *in vivo*.

Many previously published studies have shown that oxidative stress exerts critical impacts during the process of SCI [44, 45]. The definition of oxidative stress is the imbalance between oxidants (ROS and MDA) and antioxidants (GSH and SOD), which ultimately leads to cellular death [36]. ROS can cause lipid peroxidation, inactivation of proteins, and DNA fragmentation, ultimately resulting in apoptosis [46]. MDA, the ultimate product of lipid peroxidation, can directly trigger tissue damage [24]. GSH and SOD have an ability to scavenge free radicals, in order to exert antioxidative functions [47]. Consistent with results of previous research [24, 25, 48], an increase in levels of oxidative stress products (ROS and MDA) and a decrease in levels of antioxidants (GSH and SOD) were found in both the H_2_O_2_-induced BMSC and the SCI model. Treatment with CoQ10 was found to significantly decrease ROS production and increase the activity of GSH in the H_2_O_2_-induced BMSC model. *In vivo*, compared with the SCI and BMSC groups, decreasing levels of MDA and increasing levels of SOD and GSH were found in the CoQ10 treatment and combination groups. These findings indicate that CoQ10 has an ability to resist oxidative stress.

In order to investigate the detailed molecular mechanism of antioxidation and antiapoptosis, the Nrf-2/NQO-1 signaling pathway was evaluated. The Nrf-2/NQO-1 signaling pathway plays a pivotal role in antioxidative stress. Nuclear factor erythroid 2-related factor 2 (Nrf-2), a transcription factor, is involved in the regulation of a number of ROS-detoxifying enzymes. NQO-1, a widely distributed FAD-dependent flavoprotein, accelerates obligatory two-electron reductions of nitroaromatics, quinones, azo dyes, and quinonimines. NQO-1 exerts highly effective antioxidant functions and performs a cytoprotective role [49]. When oxidative stress is exerted, Nrf-2 is liberated from Keap-1 and is translocated into the nucleus, where it combines with ARE and activates the ARE-dependent transcription of phase II and antioxidant defense enzymes, including heme oxygenase-1 (HO-1) and NAD (P)H:quinone acceptor oxidoreductase 1 (NQO-1), attenuating cellular oxidative stress [50, 51]. In the present study, we found that levels of Nrf-2 and NQO-1 proteins significantly decrease in both H_2_O_2_-induced BMSC death and the SCI rat model. These findings are consistent with previous research results [23, 25, 52]. However, treatment with CoQ10 significantly increases the levels of Nrf-2 and NQO-1 proteins, both *in vitro* and *in vivo*. These results indicate that CoQ10 exerts an antioxidant and antiapoptotic function via the Nrf-2/NQO-1 signaling pathway. In order to further evaluate the involvement of the Nrf-2/NQO-1 signaling pathway, a unique inhibitor of the Nrf-2 pathway, brusatol, was used [53]. The results show that brusatol reverses the protective effect and downregulates the expression of Nrf-2/NQO-1 proteins *in vitro*. These results indicate that the Nrf-2/NQO-1 signaling pathway is involved in the antioxidant and antiapoptotic effects of CoQ10 against H_2_O_2_-induced cell death. In the future study, we will use Nrf-2 and NQO-1 KO/KI models to further confirm that CoQ10 exerts an antioxidant and antiapoptotic function via the Nrf-2/NQO-1 signaling pathway.

In conclusion, our results indicate that CoQ10 exerts an antiapoptotic and antioxidant effect against H_2_O_2_-induced BMSC death via activating the Nrf-2/NQO-1 pathway *in vitro*. Moreover, CoQ10 also decreases oxidative stress and was found to improve the microenvironment in the rat SCI model. Therefore, it may be able to protect BMSCs from oxidative stress and enhance the therapeutic efficiency of BMSC transplantation for SCI treatment.

## Figures and Tables

**Table 1:** Primers used for target amplification in this study.

Name	Primer	Accession number	Sequence (5′-3′)
Bax	Forward	NM_017059.2	CACCAGCTCTGAACAGATCA
	Reverse		ATCGCCAATTCGCCTGAGAC

Caspase-3	Forward	XM_006253130.3	CGATTATGCAGCAGCCTCAA
	Reverse		AGGAGATGCCACCTCTCCTT

Bcl-2	Forward	NM_016993.1	CTGTGGATGACTGAGTACCTGAAC
	Reverse		AGAGACAGCCAGGAGAAATCAAAC

GAPDH	Forward	XM_017592435.1	CAAGTTCAACGGCACAGTCAAG
	Reverse		ACATACTCAGCACCAGCATCAC

## Data Availability

The data used to support the findings of this study are available from the corresponding authors upon request.
